# Hepatopleural Fistula Secondary to a Persistent Liver Abscess: A Case Report

**DOI:** 10.7759/cureus.99876

**Published:** 2025-12-22

**Authors:** Juan J Vergara Torrente, Maria Camila Velasquez, Santiago Velasquez, Angelica del Carmen Narvaez Morelo

**Affiliations:** 1 Department of Medicine, Fundación Universitaria Autónoma de Las Américas, Pereira, COL; 2 Department of General Medicine, Fundación Universitaria Autónoma de Las Américas, Pereira, COL; 3 Department of General Medicine, Universidad del Sinú, Monteria, COL

**Keywords:** empyema, hepatopleural fistula, liver abscess, percutaneous drainage, thoracotomy

## Abstract

Hepatopleural fistula (HPF) is an abnormal communication between the pleural cavity and the hepatobiliary system; it is uncommon and carries relevant morbidity. Reported etiologies include thoracoabdominal trauma, liver abscess of different causes, biliary stenosis or obstruction, postsurgical iatrogenesis, and even congenital anomalies. We present the case of a 37-year-old woman with six months of right upper-quadrant pain and intermittent fever. Contrast-enhanced computed tomography (CT) showed a liver abscess; percutaneous drainage achieved initial improvement. One month later, she presented with exertional dyspnoea, productive cough, and fever. Studies demonstrated a loculated right pleural effusion and a persistent abscess, with imaging signs of hepatopleural communication suggestive of HPF. A pleural tube was placed with an initial 2,000 mL output, followed by posterolateral thoracotomy with decortication and fistula closure, plus anterior and posterior chest drainage. The course was favorable with resolution of infection and discharge with imaging follow-up. This case underscores the need for high clinical suspicion in unresolved liver abscesses progressing to empyema and supports timely surgical management in the absence of biliary obstruction, complemented by targeted antibiotics.

## Introduction

Hepatopleural fistula (HPF) is a rare entity defined by a communication between the pleura and the hepatobiliary system, traditionally reported as a complication of infectious hepatic processes, trauma, and biliary tract obstruction [1,2,3-5]. Recognition may be delayed due to clinical overlap with other causes of pleural effusion and is associated with adverse outcomes if the infectious focus persists [1,3,4].

HPF typically develops when a persistent liver abscess or elevated biliary pressure erodes the diaphragm, establishing a tract from the hepatic parenchyma/biliary tree into the pleural space, a process facilitated by the thoracoabdominal pressure gradient and local inflammation. Clinical features include bilothorax, pleural effusion most often on the right side, and cough with bilious sputum when a bronchobiliary communication is present. Nevertheless, HPF remains underrecognized and often mimics common pleural or pulmonary conditions, which frequently delays diagnosis.

Series and case reports describe different therapeutic strategies: percutaneous drainage of the liver abscess, pleural drainage with thoracentesis, video-assisted thoracoscopic surgery (VATS) or thoracotomy with decortication, and, when biliary obstruction exists, endoscopic decompression via endoscopic retrograde cholangiopancreatography (ERCP) [4-6]. We report HPF secondary to a persistent liver abscess after percutaneous drainage that required open surgical management with good outcomes.

## Case presentation

A 37-year-old woman, with no relevant medical history, presented with right upper quadrant abdominal pain for six months without clear triggers and intermittent subjective fever. She was initially evaluated by her family physician, who ordered blood tests and an upper abdominal ultrasound. Laboratory tests showed no acute phase reactants and no abnormalities in liver function tests or bilirubin levels. Ultrasound revealed an oval lesion in segment VIII with a well-defined wall, measuring approximately 500 mL, suggestive of a hepatic abscess. Given clinical stability, ultrasound-guided percutaneous drainage was performed, yielding 300 mL of purulent material, and culture-directed antibiotic therapy was initiated. Over the following days, drainage remained around 50 mL per day; in the absence of symptoms and with decreased purulence, the catheter was removed.

**Figure 1:**
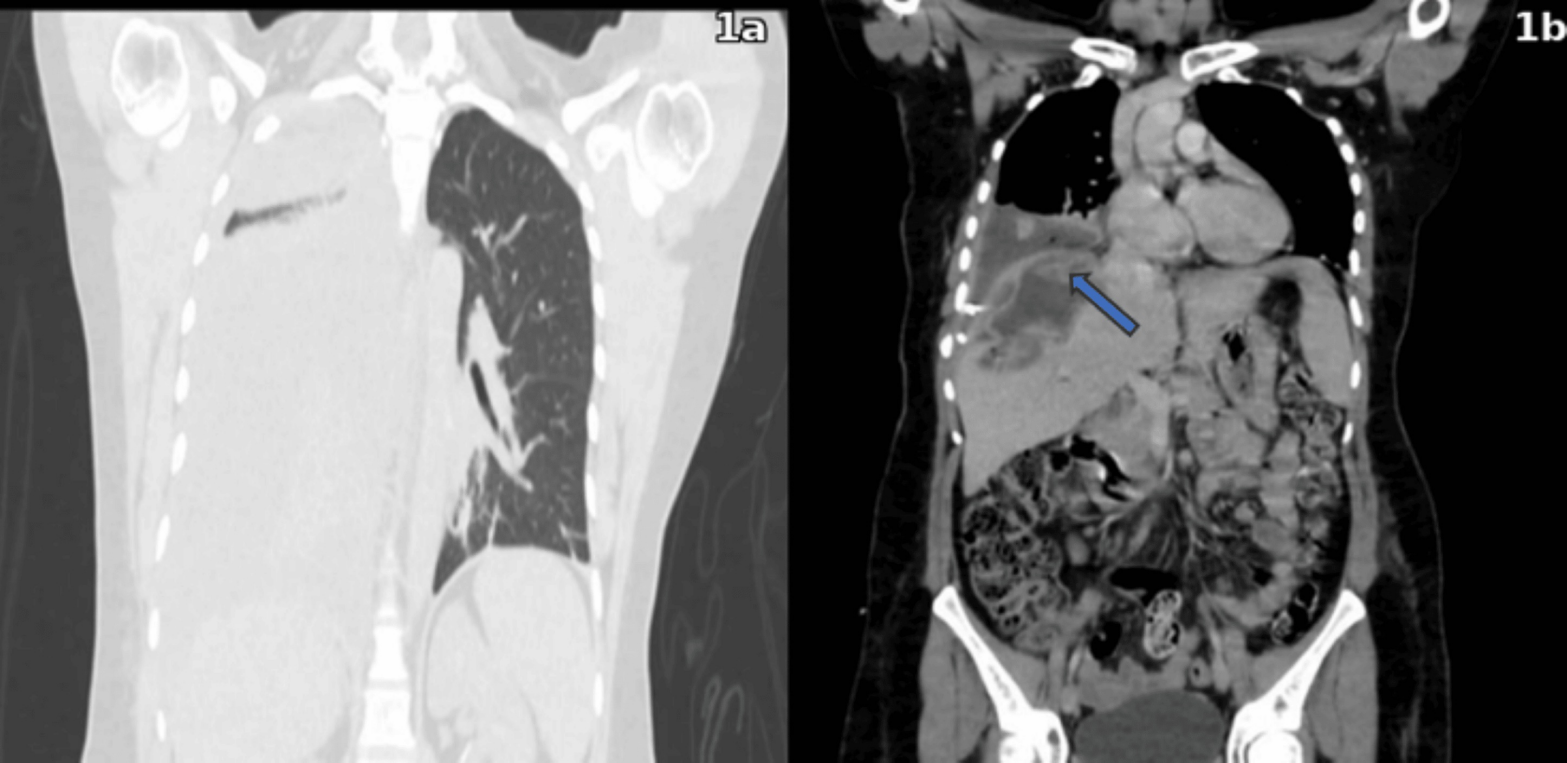
Contrast-enhanced chest CT 1a. Coronal section showing a right pleural effusion; 1b. Coronal section demonstrating a communication between the diaphragm and the right hepatic lobe, suggestive of a hepatopleural fistula (blue arrow).

One month later, she experienced a recurrence of symptoms characterized by abdominal pain, progressively worsening exertional dyspnea, productive cough, and fever, suggesting persistence or complication of the underlying infection. On physical examination, lung auscultation revealed coarse crackles at the right lung base. The abdomen was soft, with mild deep tenderness to palpation in the right upper quadrant, without peritoneal signs. Admission laboratory studies demonstrated leukocytosis with neutrophil predominance, elevated C-reactive protein, abnormalities in liver function tests, and hyperbilirubinemia (Table 1). Chest radiography showed a right pleural effusion. Contrast-enhanced thoracoabdominal computed tomography (CT) confirmed a loculated right pleural effusion occupying approximately 50% of the hemithorax (Figure 1a), a hepatic abscess in segments VII and VIII, and imaging findings suggestive of a hepatodiaphragmatic communication consistent with an HPF (Figure 1b).

**Table 1:** Admission values and laboratory-specific reference ranges

Serum Parameter	Measured Value	Reference Range
White blood cell	19 300 k/µL	4.0–11.0 k/µL
Neutrophils	92.2 %	37–80 %
C-reactive protein	15 mg/dL	0.0–1.0 mg/dL
Alanine aminotransferase	48 U/L	10–42 U/L
Aspartate aminotransferase	49 U/L	10–42 U/L
Total bilirubin	2.2 mg/dL	0.2–1.0 mg/dL

A right pleural drainage tube was placed, with 2,000 mL of purulent fluid drained in the first 24 hours. Based on the anatomical characteristics, pleural thickening, and our team’s experience, a right posterolateral thoracotomy with decortication was selected as the surgical approach, along with fistula closure and placement of two pleural drains.

**Figure 2:**
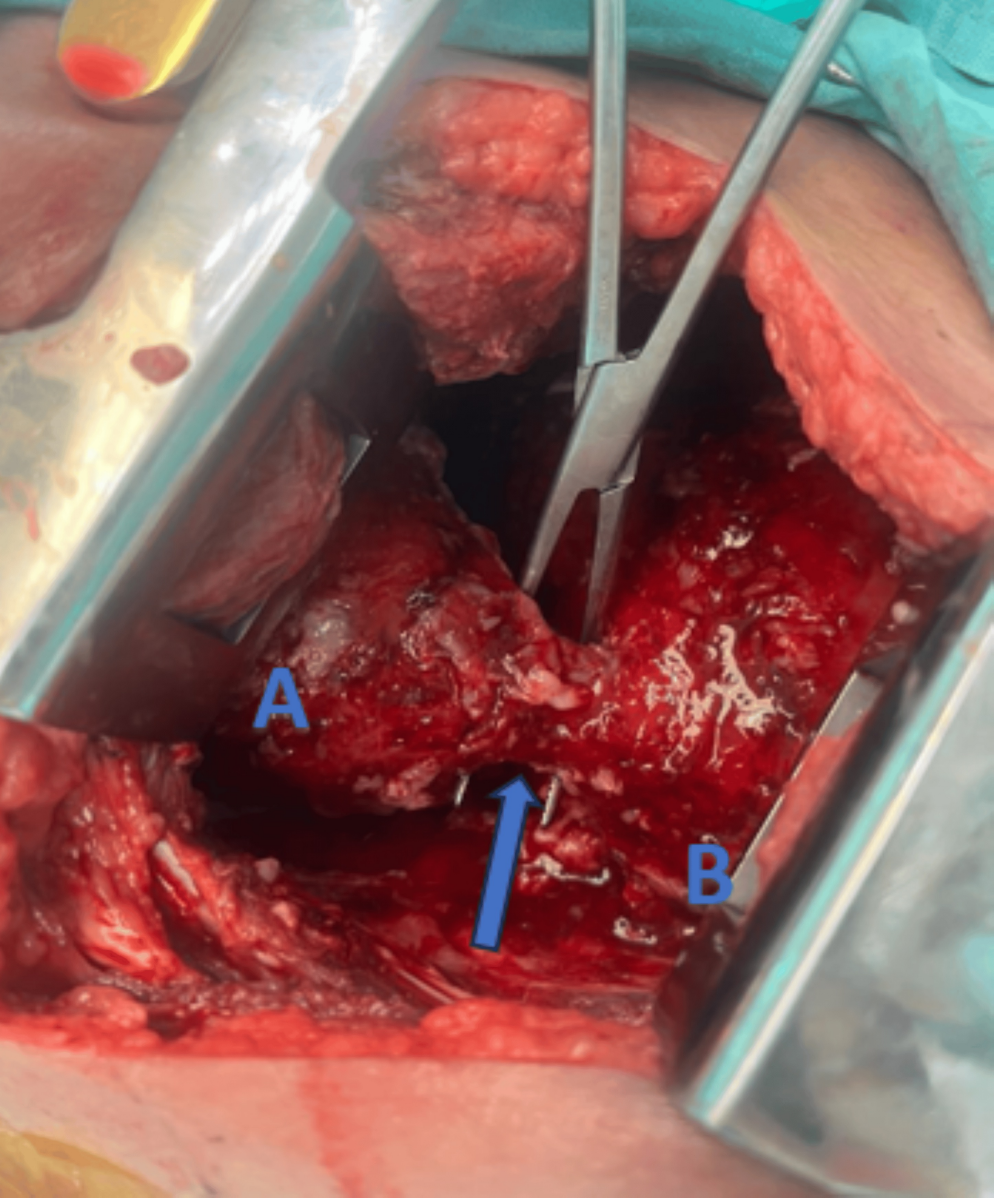
Intraoperative findings The blue arrow indicates the fistulous tract; A denotes the lower lung lobe; B denotes the diaphragmatic dome.

Intraoperatively, pleural thickening of at least 1 cm with fibrinopurulent empyema and a fistulous tract extending from the right diaphragmatic dome to the ipsilateral lower lobe were observed (Figure 2). The culture of the drained fluid grew *Escherichia coli*; targeted antibiotic therapy with piperacillin-tazobactam was initiated. The percutaneous abdominal drain was reinserted (output 100 mL/day), and the pleural drain was maintained (400 mL/day), both showing a progressive decline. After a joint evaluation with the hepatobiliary and thoracic surgery services, the pleural and abdominal drains were removed on postoperative day seven. The patient was discharged in good condition with follow-up; abdominal CT showed no evidence of hepatopleural fistula or pleural effusion and no hepatic abscess.

## Discussion

HPF is an abnormal communication between the biliary tree or hepatic parenchyma and the pleural space. It usually arises in the setting of persistent hepatic infection, especially pyogenic abscesses, with *Klebsiella *as a frequent pathogen, as well as after diaphragmatic or hepatic injury due to trauma or surgical interventions. It may also be associated with biliary tract obstruction and, less commonly, with tumorous infiltration (e.g., hepatocellular carcinoma) or congenital anomalies; advanced liver disease, such as portal hypertension and cirrhosis, can coexist [7]. Physiopathologically, the thoracoabdominal pressure gradient and local inflammation promote fistulous-tract formation, resulting in biliothorax or right-sided empyema [2, 8, 9].

Management should prioritize source control: pleural and abscess drainage with culture-directed antibiotics. Where biliary obstruction or intraductal hypertension is present, endoscopic decompression via ERCP may hasten clinical resolution. In the setting of loculated effusions, pleural fibrosis, or failure of conservative measures, thoracic intervention, either VATS or open thoracotomy, allows decortication and fistula closure with high success rates; the choice depends on team experience and anatomical complexity. Timely source control and technical expertise are pivotal to outcomes [2,4-6,10]. This case illustrates HPF secondary to a persistent liver abscess with progression to empyema and the need for definitive surgical intervention despite initial percutaneous drainage.

We adopted a staged, mixed approach: an initial open thoracic procedure for empyema control, decortication, and fistula closure, followed by a second percutaneous abdominal intervention for complementary hepatic drainage, plus targeted antibiotics. Indications were based on the persistent hepatic focus and refractory loculated effusion limiting pulmonary re-expansion and perpetuating local sepsis. Early intervention enabled effective source control, progressive decline in outputs, timely drain removal, and an uncomplicated discharge, consistent with contemporary recommendations for complicated HPF.

The literature indicates that HPF commonly arises from persistent or unresolved liver abscesses and that, in the setting of loculated pleural collections, pleural fibrosis, or failure of initial percutaneous drainage, thoracic interventions (VATS or open thoracotomy with decortication) and direct closure of the fistulous tract, combined with drainage of the hepatic source and culture-directed antibiotics, are associated with improved outcomes; furthermore, when biliary hypertension or obstruction is present, endoscopic decompression can expedite recovery. In our case, clinical relapse after percutaneous drainage, a loculated right empyema, and the absence of biliary obstruction prompted a staged approach consisting of decortication and fistula closure plus complementary hepatic drainage and targeted antimicrobial therapy, yielding a favorable course consistent with these recommendations.

## Conclusions

HPF is an infrequent complication of liver abscess with potential progression to empyema. Timely diagnosis requires a high index of suspicion and imaging correlation. Treatment selection should be individualized according to the predominant pathophysiology, raised biliary pressure versus a direct hepatopleural communication, ranging from biliary decompression to decortication and fistula closure, where a loculated effusion and a persistent infectious focus predominate. A multidisciplinary and early approach is associated with better outcomes. In addition, microbiological confirmation and targeted antibiotic therapy optimize infection control, while postoperative clinical and imaging surveillance allow early detection and treatment of recurrences or residual collections. This case supports a stepwise algorithm (drainage, source control, and, where necessary, surgical intervention). Although a single report, the findings align with available evidence and support early evaluation by thoracic and hepatobiliary teams to reduce morbidity and accelerate recovery.
